# Association Between the Characteristics of mHealth Apps and User Input During Development and Testing: Secondary Analysis of App Assessment Data

**DOI:** 10.2196/46937

**Published:** 2023-11-22

**Authors:** Anna-Lena Frey, Rebecca Baines, Sophie Hunt, Rachael Kent, Tim Andrews, Simon Leigh

**Affiliations:** 1 Organisation for the Review of Care and Health Apps Daresbury United Kingdom; 2 Centre for Health Technology University of Plymouth Plymouth United Kingdom; 3 Department of Digital Humanities King’s College London London United Kingdom; 4 Warwick Medical School University of Warwick Coventry United Kingdom

**Keywords:** patient and public involvement, user involvement, mobile apps, digital health, mobile health, quality assessment

## Abstract

**Background:**

User involvement is increasingly acknowledged as a central part of health care innovation. However, meaningful user involvement during the development and testing of mobile health apps is often not fully realized.

**Objective:**

This study aims to examine in which areas user input is most prevalent and whether there is an association between user inclusion and compliance with best practices for mobile health apps.

**Methods:**

A secondary analysis was conducted on an assessment data set of 1595 health apps. The data set contained information on whether the apps had been developed or tested with user input and whether they followed best practices across several domains. Background information was also available regarding the apps’ country of origin, targeted condition areas, subjective user ratings, download numbers, and risk (as per the National Institute for Health and Care Excellence Evidence Standards Framework [ESF]). Descriptive statistics, Mann-Whitney *U* tests, and Pearson chi-square analyses were applied to the data.

**Results:**

User involvement was reported by 8.71% (139/1595) of apps for only the development phase, by 33.67% (537/1595) of apps for only the testing phase, by 21.88% (349/1595) of apps for both phases, and by 35.74% (570/1595) of apps for neither phase. The highest percentage of health apps with reported user input during *development* was observed in Denmark (19/24, 79%); in the condition areas of diabetes (38/79, 48%), cardiology (15/32, 47%), pain management (20/43, 47%), and oncology (25/54, 46%); and for high app risk (ESF tier 3a; 105/263, 39.9%). The highest percentage of health apps with reported user input during *testing* was observed in Belgium (10/11, 91%), Sweden (29/34, 85%), and France (13/16, 81%); in the condition areas of neurodiversity (42/52, 81%), respiratory health (58/76, 76%), cardiology (23/32, 72%), and diabetes (56/79, 71%); and for high app risk (ESF tier 3a; 176/263, 66.9%). Notably, apps that reported seeking user input during testing demonstrated significantly more downloads than those that did not (*P*=.008), and user inclusion was associated with better compliance with best practices in clinical assurance, data privacy, risk management, and user experience.

**Conclusions:**

The countries and condition areas in which the highest percentage of health apps with user involvement were observed tended to be those with higher digital maturity in health care and more funding availability, respectively. This suggests that there may be a trade-off between developers’ willingness or ability to involve users and the need to meet challenges arising from infrastructure limitations and financial constraints. Moreover, the finding of a positive association between user inclusion and compliance with best practices indicates that, *where no other guidance is available*, users may benefit from prioritizing health apps developed with user input as the latter may be a proxy for broader app quality.

## Introduction

### Background

User involvement, also referred to as patient and public involvement, is increasingly being acknowledged as a central part of health care innovation [1]. In recent years, user involvement policies and strategies have been developed by organizations such as the National Institute for Health and Care Excellence (NICE) [2], the UK National Health Service [3,4], and the US Food and Drug Administration [5] to ensure that patient and public input is actively taken into account during the research and development of medical guidelines, products, and services.

A key social and political driver behind the increased emphasis placed on user involvement is the recognition that health care transformation is crucial to addressing current challenges and that, for such transformation to be efficient and successful, a people-centered approach needs to be taken [3,6]. For instance, the pressures faced by health care systems as a result of an increase in the number of older adult patients may be partially eased through more widespread use of health technologies and data. However, to achieve a positive impact, technologies have to address patient needs and must be easy to use, which can arguably only be accomplished by involving the public in the design of such innovations [6]. Another important challenge that has become particularly apparent during the COVID-19 pandemic is inequality in health care [7]. To ensure that new health technologies help improve rather than exacerbate such inequalities, it is critical to actively involve disadvantaged individuals to understand their needs and the barriers they may face to accessing health innovations [3]. In addition, shifts in demographics, such as an aging population and an increase in migration, highlight the need to seek input from diverse groups of individuals to ensure that their potential concerns related to new technologies are considered [6]. If such a participatory approach to health care innovation is taken, this can lead to more efficient use of resources and higher product uptake [3,8,9], as well as to the empowerment of patients to be active partners in decisions affecting their care [9,10].

However, despite these benefits, co-design and other forms of user involvement during the development and testing of mobile health apps are often not regarded as essential or are not fully realized [11-13]. Some developers express support for the idea of user involvement without implementing it, whereas others do not follow sound methodologies or do not meaningfully involve users throughout the app life cycle [11-13]. Moreover, remote user involvement, which has been on the rise since the beginning of the COVID-19 pandemic, brings with it additional implementation challenges related to technology access, digital literacy, data privacy, and difficulties in “claiming space” to speak out during web-based meetings that may not always be adequately addressed [14-16].

Given the reported benefits of meaningfully involving users, it is important to understand in which areas user inclusion is currently being implemented during the development and testing of mobile health apps. Such insights can help identify areas in which more education and support are needed to encourage app developers to seek user input. Moreover, an understanding of the possible relationships among user involvement, compliance with best practices, and app use and acceptance (eg, as indicated by user ratings and download numbers) can provide valuable guidance for future innovation practices.

### Objectives

Therefore, the aims of this study were 3-fold: first, to examine how, if at all, the prevalence of user input during health app development and testing differs across countries, condition areas, and app risks; second, to determine whether user involvement is associated with higher user ratings or app download numbers; and, finally, to assess whether user input is associated with better compliance with best practices across the domains of clinical assurance, data privacy, risk management, and user experience. Exploring such associations may help strengthen the case for stakeholders, including patients and health care professionals, to prioritize mobile health apps that included users during their development and testing as this may be a proxy for broader app quality.

## Methods

### Data Provenance and Characteristics

A secondary analysis was conducted on a data set containing background and assessment information on 1595 mobile health apps. The data were collected between January 2021 and January 2022 as part of an app review conducted by the Organisation for the Review of Care and Health Apps (ORCHA), a digital health compliance company that specializes in the evaluation of mobile health apps and is currently working with National Health Service providers across 70% of regions in England [17].

During the ORCHA Baseline Review (OBR; version 6), each app was evaluated using approximately 300 objective (mostly binary yes or no) questions. The initial evaluation was performed by 1 of the ORCHA assessors, all of whom had undergone a thorough 6-month training course on standard operating procedures for the OBR and perform health app evaluations on a daily basis as part of their job. The OBR responses for all 1595 apps were reviewed and signed off by a second more senior assessor. Any disagreements between the 2 assessors were resolved by involving a third assessor with extensive review experience or, in the case of more difficult issues, were discussed by a panel of subject matter experts in the areas of assessment, clinical practice, and research who resolved the matter through a consensus decision. Notably, the OBR questions were based on standards, guidelines, and regulatory requirements such as the UK Medicines and Healthcare Products Regulatory Agency guidance on software as a medical device, the European Union General Data Protection Regulation (GDPR), the Web Content Accessibility Guidelines, the UK National Health Service DCB0129 Clinical Risk Management standard, and the NICE Evidence Standards Framework (ESF; see further details in the following paragraphs). As such, the OBR questions reflect widely accepted best practices.

For this study, a subset of 14 assessment questions from the OBR was selected for analysis based on their hypothesized association with user involvement. The questions covered best practices in the following domains: clinical assurance, data privacy, risk management, and user experience. The exact phrasing of the questions is noted in the *Results* section.

As part of the assessment, mobile health apps were classified into different tiers following the NICE ESF [18]. Tiers were assigned to the apps based on their functionality, which has implications for the risk and evidentiary requirements of the apps. The following ESF definitions were applied (note that the ESF has since been updated, with tiers 1, 2, and 3a or 3b having been replaced by tiers A, B, and C, respectively [19]):

1. Tier 1: apps that provide health and social care services with no measurable user outcomes
2. Tier 2: apps that provide 2-way communication between users and health care professionals, provide health information, or offer a health diary
3. Tier 3a: apps that support preventative behavior change aimed at health issues or allow users to self-manage a specific condition
4. Tier 3b: apps that provide or guide treatment for a condition; record and transmit data about this condition to a health care professional, carer, or third party without the user’s input; contain a calculator that affects treatment; or guide diagnosis [18]

In addition to the ESF tier, the following background information was collected for each app: country of origin, app store user rating, downloads, and targeted condition areas. Note that download numbers were only available from the Google Play Store (for Android apps; n=777) and not from the iOS App Store. Moreover, a given app could cover more than one condition area.

### Determination of User Input

To classify mobile health apps into those that did and did not report seeking user input during development or testing, 2 OBR assessment questions were used. These questions are listed below together with the conditions under which they were answered with *yes* (thus indicating user input).

#### Question 1: Is There a Statement Within the App or Store About User Feedback During Design or Development?

This question was answered with *yes* if information within the app or an associated policy or website stated that the app (1) was changed based on feedback received from users (eg, through suggestion forms provided on the associated website), (2) underwent a survey or pilot study and changes were made based on the outcome, or (3) was designed by the developer or publisher as a remedy to a problem that they (or someone they were caring for) were experiencing (that is to say, the developer was part of the intended user group and, therefore, had a firsthand understanding of user needs).

#### Question 2: Is There a Statement Within Either the App or Store About User Input During Testing?

This question was answered with “yes” if information within the app or an associated policy or website mentioned (1) a case study for the app, (2) that a beta version of the app was available before the app went live, (3) user feedback stating that the app is beneficial, (4) evidence of indicated user benefits, or (5) any other evidence of user testing.

### Statistical Analysis

Descriptive statistics were used to examine the prevalence of user input for mobile health apps across different countries, condition areas, and ESF tiers. Note that only countries and condition areas with >10 apps were included in the relevant data summaries as samples of <10 apps are likely not representative of the larger app “population” in a given country or condition area.

Mann-Whitney *U* tests were conducted to assess differences in user ratings and download levels between mobile health apps that did and did not report seeking user input during development or testing. The Mann-Whitney *U* test is a nonparametric test used to compare ordinal or nonnormally distributed continuous dependent variables between 2 independent groups. Test statistics are calculated by placing the values of the dependent variable in ascending order (disregarding group membership); assigning a rank to each value; and then using the group sum of ranks and the group sample sizes to calculate the *U* value, the *Z* statistic, and an associated *P* value (for further details, see the book by Field [20]). We used Mann-Whitney tests as Shapiro-Wilks tests indicated that the user rating data were not normally distributed and as the download data were ordinal. Specifically, only download *ranges* were available from the app store (eg, 1-4, 5-9, 10-49, 50-99, and 100-499 up to 1 billion downloads), and each range was treated as a separate ordinal category in the analysis, referred to in the following sections as “download levels” (see Multimedia Appendix 1 for details).

Furthermore, Pearson chi-square analyses were performed to examine bivariate associations between user input and specific quality indicators of mobile health apps (as captured by individual dichotomous assessment questions), and odds ratios were reported. The Pearson chi-square test was used to examine relationships between 2 categorical variables, which can be represented in an *i* by *j* table, with *i* designating the number of categories in the first variable and *j* the number of categories in the second variable. The chi-square value represents the sum of squares of SDs between the *observed* frequencies within each cell of the table and the *expected* frequencies in each cell, with the latter being determined based on the total number of observations for the different categories. By comparing the calculated chi-square value against the critical values of the known chi-square distribution, considering the *df*, a *P* value can be obtained (for further details, see the book by Field [21]).

Unless otherwise indicated, the reported results remained significant after multiple-comparison correction for the 28 conducted association analyses (user involvement during testing and development examined for 14 questions) using the Benjamini-Hochberg method with a false discovery rate of 10%. The Benjamini-Hochberg method is a multiple-comparison correction method that controls the false discovery rate. As part of this method, *P* values are placed in ascending order; a rank is assigned to each *P* value; and a Benjamini-Hochberg critical value is calculated for each *P* value using the rank, total number of tests, and selected false discovery rate. All *P* values above (but not below) the largest *P* value that is smaller than its critical value are considered significant (for further details, see the work by Benjamini and Hochberg [22]).

All analyses were conducted using SPSS Statistics (version 27.0; IBM Corp). Statistical significance was defined at the usual 5% level (ie, *P*<.05).

### Ethical Considerations

As part of the OBR process, developers are informed of their assessment results and given the opportunity to contest the findings and request an amendment based on additional information. Moreover, the ORCHA privacy policy states that all reviews can be used for research purposes unless the developer asks for their app to be removed from the research database. Furthermore, outputs were anonymized in this paper, with no individual mobile health apps being named. As no data from human participants was used in this study, ethical approval was not required.

## Results

### App Characteristics

**Figure 1:**
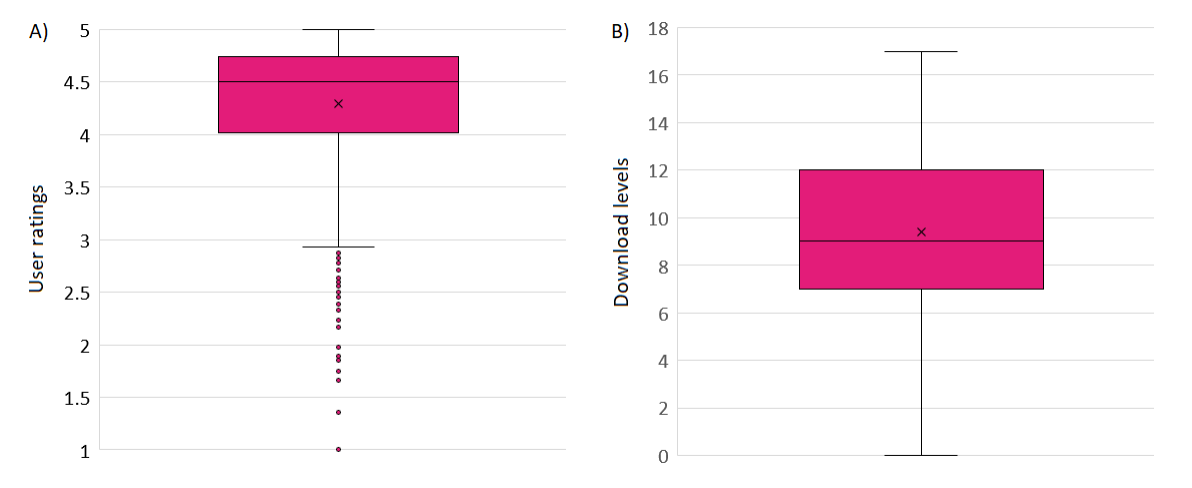
Distribution of (A) user ratings and (B) download levels across all mobile health apps. Apps with a higher download level have a higher number of downloads, as indicated by the range of downloads shown in the app store (see the Methods section for details).

The number of mobile health apps within each country, condition area, and ESF tier can be found in Tables 1-3 and the distribution of user ratings and download levels across all apps is presented in Figure 1. Within the current data set, the largest number of health apps was developed in the United States (490/1595, 30.72%) and the United Kingdom (419/1595, 26.27%). The most covered condition areas were healthy living (563/1595, 35.3%), mental health (440/1595, 27.59%), and neurological conditions (135/1595, 8.46%).

Overall, user involvement was reported by 8.71% (139/1595) of the apps for only the development phase, by 33.67% (537/1595) of the apps for only the testing phase, by 21.88% (349/1595) of the apps for both phases, and by 35.74% (570/1595) of the apps for neither phase.

**Table 1:** Number and percentage of mobile health apps across countries that reported seeking user input during development and testing.

Country of origin	Apps with user input during development, n (%)	Apps with user input during testing, n (%)
Denmark (n=24)	19 (79.2)	17 (70.8)
United Kingdom (n=419)	185 (44.2)	255 (60.9)
France (n=16)	7 (43.8)	13 (81.3)
Turkey (n=14)	6 (42.9)	9 (64.3)
Canada (n=71)	30 (42.3)	38 (53.5)
Belgium (n=11)	4 (36.4)	10 (90.9)
Netherlands (n=30)	10 (33.3)	21 (70)
Israel (n=22)	7 (31.8)	16 (72.7)
China (n=10)	3 (30)	6 (60)
Spain (n=24)	7 (29.2)	11 (45.8)
Poland (n=11)	3 (27.3)	1 (9.1)
Sweden (n=34)	9 (26.5)	29 (85.3)
Germany (n=51)	12 (23.5)	33 (64.7)
United States (n=490)	113 (23.1)	274 (55.9)
Australia (n=36)	8 (22.2)	15 (41.7)
Ireland (n=19)	4 (21.1)	14 (73.7)
India (n=43)	2 (4.7)	14 (32.6)
Russia (n=16)	0 (0)	3 (18.8)
Singapore (n=20)	0 (0)	8 (40)

**Table 2:** Number and percentage of mobile health apps across condition areas that reported seeking user input during development and testing^a^.

Targeted condition or group	Apps with user input during development, n (%)	Apps with user input during testing, n (%)
Diabetes (n=79)	38 (48.1)	56 (70.9)
Cardiology (n=32)	15 (46.9)	23 (71.9)
Pain management (n=43)	20 (46.5)	29 (67.4)
Cancer (n=54)	25 (46.3)	32 (59.3)
Neurological (n=135)	52 (38.5)	77 (57)
Respiratory (n=76)	29 (38.2)	58 (76.3)
Gastrointestinal (n=24)	9 (37.5)	12 (50)
Musculoskeletal (n=53)	19 (35.8)	35 (66)
Child health (n=71)	24 (33.8)	45 (63.4)
Pregnancy (n=81)	25 (30.9)	43 (53.1)
Allergy (n=13)	4 (30.8)	7 (53.8)
Neurodiverse (n=52)	15 (28.8)	42 (80.8)
Women’s health (n=66)	19 (28.8)	35 (53)
Mental health (n=440)	126 (28.6)	273 (62)
Dental (n=25)	7 (28)	13 (52)
Health living (n=563)	137 (24.3)	316 (56.1)
Dermatology (n=29)	7 (24.1)	12 (41.3)
Otorhinolaryngology (n=25)	5 (20)	9 (36)
Ophthalmology (n=56)	10 (17.9)	25 (44.6)
Sexual health (n=57)	9 (15.8)	29 (50.9)
First aid (n=13)	1 (7.7)	5 (38.5)
Urology (n=15)	1 (6.7)	10 (66.7)

^a^Note that a given app could cover more than 1 condition area.

**Table 3:** Number and percentage of mobile health apps that reported seeking user input during development or testing by National Institute for Health and Care Excellence Evidence Standards Framework (ESF) tier.

ESF tier	Apps with user input during development, n (%)	Apps with user input during testing, n (%)
Tier 1 (n=19)	3 (15.8)	5 (26.3)
Tier 2 (n=1207)	352 (29.2)	637 (52.8)
Tier 3a (n=263)	105 (39.9)	176 (66.9)
Tier 3b (n=105)	28 (26.7)	68 (64.8)

### User Input Across Countries, Condition Areas, and ESF Tiers

Examining user input across countries revealed that mobile health apps originating from Denmark had the highest percentage of reported user inclusion during development (19/24, 79%), followed by apps developed in the United Kingdom (185/419, 44.2%), France (7/16, 44%), Turkey (6/14, 43%), and Canada (30/71, 42%; Table 1). The percentage of mobile health apps reporting seeking user input during testing was highest in Belgium (10/11, 91%), Sweden (29/34, 85%), and France (13/16, 81%; Table 1). Across all countries except Denmark, the percentage of apps that included users during development was lower than the percentage of apps seeking user input during testing.

When considering apps across different health conditions, it was observed that the areas of diabetes (38/79, 48%), cardiology (15/32, 47%), pain management (20/43, 47%), and oncology (25/54, 46%) contained the highest percentage of apps that reported including users during development (Table 2). The percentage of apps reporting seeking user input during testing was highest for the condition areas of neurodiversity (42/52, 81%), respiratory health (58/76, 76%), cardiology (23/32, 72%), and diabetes (56/79, 71%; Table 2). Across all condition areas, the percentage of apps that included users during development was lower than the percentage of apps seeking user input during testing.

When examining mobile health apps across different ESF tiers, it was found that, with an increasing ESF tier (and, thus, with higher risk), an increasing percentage of apps reported seeking user input during development or testing. The exception to this pattern was tier 3b, in which a smaller percentage of apps reported including users during development compared with tiers 3a and 2. The percentage of apps reporting user input during testing was also slightly smaller for tier 3b than for tier 3a (Table 3).

### User Input, User Ratings, and Download Levels

Mann-Whitney *U* tests indicated that the distribution of user ratings did not differ significantly between mobile health apps that did and did not report seeking user input during development (*P*=.45) or testing (*P*=.27).

**Figure 2:**
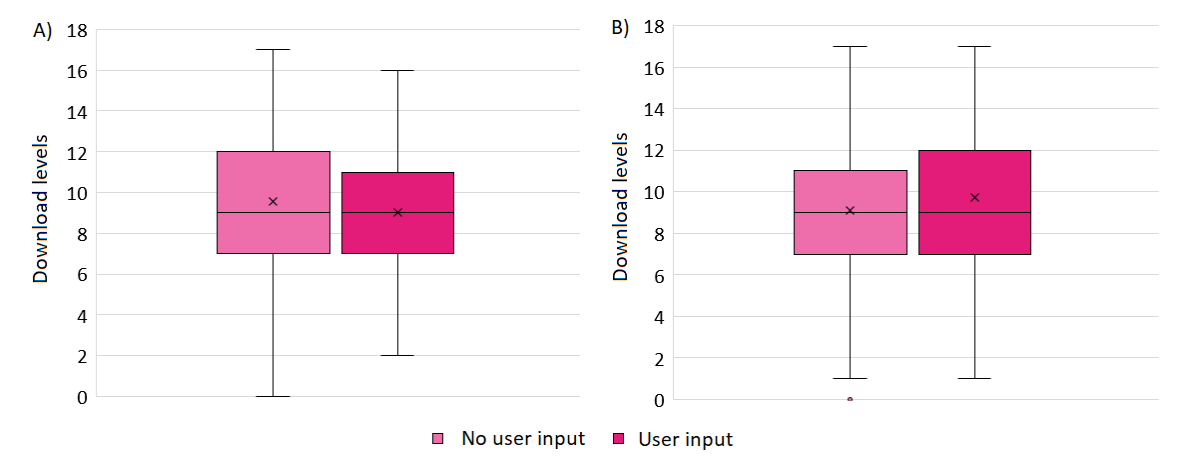
Download levels for mobile health apps that did and did not report seeking user input during (A) development or (B) testing. Apps with a higher download level have a higher number of downloads, as indicated by the range of downloads shown in the app store (see the Methods section for details).

By contrast, the distribution of download levels demonstrated a significant difference for mobile health apps that did and did not report including users during development (*P*=.02) or testing (*P*=.008). However, the direction of this effect differed between the 2 comparisons: apps that did not report including users *during development* showed a *larger* mean rank (and, thus, more downloads) than those that reported including users, whereas apps that did not report seeking user input *during testing* demonstrated a *smaller* mean rank (and, thus, fewer downloads) than those that did report seeking user input (Figure 2).

### User Input and App Compliance With Best Practices

#### Overview

Significant associations between compliance with best practices, as assessed through selected OBR questions, and reported user input were observed across all domains (clinical assurance, data privacy, risk management, and user experience).

The results of the Pearson chi-square analyses are described in detail in the following sections.

#### Clinical Assurance

Pearson chi-square analysis revealed a significant association between the answer to the question—“Does the app have appropriate evidence for its ESF tier?”—and the reported involvement of users during development (*χ*^2^_1_=24.8, *P*<.001) and testing (*χ*^2^_1_=218.2, *P*<.001). The odds ratio indicated that, if a mobile health app reported seeking user input during development or testing, the odds of it meeting its tier’s evidence requirements were 1.75 (95% CI 1.40-2.19) or 6.02 (95% CI 4.68-7.75) times higher, respectively, than if it did not include users.

When only considering health apps for which an efficacy or effectiveness study had been conducted, no significant association was found between the significance of the study results (ie, the answer to the question “Does the p-value demonstrate significance [*P*<.05]?”) and reported user input during development (*χ*^2^_1_=0.4, *P*=.55) or testing (*χ*^2^_1_=3.0, *P*=.09).

#### Data Privacy

In the data privacy domain, a significant association was observed between the answer to the question—“Is there a policy or statement that confirms the app’s compliance with the GDPR?”—and reported user input during development (*χ*^2^_1_=22.1, *P*<.001) and testing (*χ*^2^_1_=67.0, *P*<.001). The odds ratio indicated that, if a mobile health app reported seeking user input during development or testing, the odds of it confirming compliance with the GDPR were 1.75 (95% CI 1.39-2.22) or 2.57 (95% CI 2.04-3.23) times higher, respectively, than if it did not involve users.

A significant association was also found between the answer to the question—“Does the developer provide users with details on all the purposes of processing user data?”—and the reported inclusion of users during development (*χ*^2^_1_=6.0, *P*=.01) and testing (*χ*^2^_1_=46.9, *P*<.001). The odds of explaining all data processing purposes were 1.84 (95% CI 1.12-3.01) or 4.29 (95% CI 2.74-6.72) times higher if a health app reported involving users during development or testing, respectively, than if it did not.

#### Risk Management

Regarding risk management, a significant association was revealed between the answer to the question—“Does the developer make clear risks associated with using the app?”—and the reported inclusion of users during testing (*χ*^2^_1_=14.6, *P*<.001) but not during development (*χ*^2^_1_=0.6, *P*=.45). If a health app reported seeking user input during testing, the odds of the developer clearly stating the risks associated with using the app were 2.42 (95% CI 1.52-3.86) times higher than if it did not include users.

Furthermore, a significant association was observed between the answer to the question—“Does the developer clearly identify who the app should or should not be used by?”—and reported user input during development (*χ*^2^_1_=38.7, *P*<.001) and testing (*χ*^2^_1_=17.9, *P*<.001). The odds ratio indicated that the odds of the developer clearly stating the target group for the health app were 2.30 (95% CI 1.76-3.00) or 1.61 (95% CI 1.29-2.00) times higher if the app reported including users during development and testing, respectively, than if it did not.

Moreover, a significant association was found between the answer to the question—“Is there a way for the user to confirm that the data input is accurate?”—and the reported inclusion of users during testing (*χ*^2^_1_=5.5, *P*=.02; note that this result does not survive multiple-comparison correction) but not during development (*χ*^2^_1_=0.4, *P*=.53). The odds of a health app incorporating data accuracy checks were 1.40 (95% CI 1.06-1.86) times higher if the app involved users during testing than if it did not.

#### User Experience and App Functionality

In the user experience and app functionality domain, a significant association was observed between the answer to the question—“Does the user have options to manage the settings for push or email notifications within the app for convenience and privacy?”—and reported user input during testing (*χ*^2^_1_=7.5, *P*=.01) but not development (*χ*^2^_1_=2.5, *P*=.12). The odds ratio indicated that, if an app reported including users during testing, the odds of it allowing users to manage notifications were 1.45 (95% CI 1.11-1.89) times higher than if it did not involve users.

A significant association was also found between the answer to the question—“Are any clinical or technical terms used explained clearly to the user?”—and the reported inclusion of users during development (*χ*^2^_1_=29.2, *P*<.001) and testing (*χ*^2^_1_=57.1, *P*<.001). The odds of clearly explaining technical terms were 2.21 (95% CI 1.65-2.96) and 2.51 (95% CI 1.97-3.20) times higher if a health app reported engaging users during development and testing, respectively, than if it did not.

Furthermore, a significant association was found between the answer to the question—“Is there any statement within the app about the developer’s commitment to addressing problems reported to them (e.g., commitment to eradicate reported bugs and faults)?”—and the reported involvement of users during development (*χ*^2^_1_=37.1, *P*<.001) and testing (*χ*^2^_1_=60.5, *P*<.001). If a health app reported seeking user input during development or testing, the odds of the developer being committed to addressing reported issues were 2.19 (95% CI 1.70-2.82) or 3.02 (95% CI 2.27-4.02) times higher, respectively, than if it did not include users.

In addition, a significant association was revealed between the answer to the question—“Does the app provide gamification or goal setting features for the user?”—and the reported inclusion of users during testing (*χ*^2^_1_=74.5, *P*<.001) but not during development (*χ*^2^_1_=3.0, *P*=.08). The odds of a health app including gamification or goal setting were 2.53 (95% CI 2.04-3.13) times higher if the app involved users during testing than if it did not.

Moreover, a significant association was observed between the answer to the question—“Are there opportunities to link with other users (buddying, forums or group education)?”—and reported user input during development (*χ*^2^_1_=5.2, *P*=.02; note that this result does not survive multiple-comparison correction) or testing (*χ*^2^_1_=34.8, *P*<.001). If a health app reported including users during development or testing, the odds of the app allowing users to link to each other were 1.32 (95% CI 1.04-1.67) and 2.00 (95% CI 1.58-2.52) times higher, respectively, than if it did not involve users.

No significant association was found between the answer to the question—“Does the app allow the monitoring of key health information?”—and the reported inclusion of users during development (*χ*^2^_1_=0.0, *P*=.85) or testing (*χ*^2^_1_=2.5, *P*=.11).

## Discussion

### Principal Findings

This study aimed to examine the relationship between mobile health app characteristics and the inclusion of users during development and testing. For this purpose, a secondary analysis was conducted on an assessment data set of 1595 mobile health apps collected by ORCHA between January 2021 and January 2022. Descriptive statistics were used to explore the prevalence of user input for apps across different countries, condition areas, and ESF tiers. In addition, Mann-Whitney *U* tests and Pearson chi-square analyses were conducted to examine group differences and associations between health apps that did and did not report seeking user input and download numbers; user ratings; and assessment measures across the domains of clinical assurance, data privacy, risk management, user experience and app functionality.

Overall, user involvement was reported by 8.71% (139/1595) of the apps for only the development phase, by 33.67% (537/1595) of the apps for only the testing phase, and by 21.88% (349/1595) of the apps for both phases. The remaining 35.74% (570/1595) of the apps did not report including users during either phase. The highest percentage of mobile health apps with reported user input during *development* was observed in Denmark (19/24, 79%); in the condition areas of diabetes (38/79, 48%), cardiology (15/32, 47%), pain management (20/43, 47%), and oncology (25/54, 46%); and for high app risk (ESF tier 3a; 105/263, 39.9%). The highest percentage of health apps with reported user input during *testing* was observed in Belgium (10/11, 91%), Sweden (29/34, 85%), and France (13/16, 81%); in the condition areas of neurodiversity (42/52, 81%), respiratory health (58/76, 76%), cardiology (23/32, 72%), and diabetes (56/79, 71%); and for high app risk (ESF tier 3a; 176/263, 66.9%).

Moreover, health apps that reported seeking user input during testing demonstrated significantly more downloads than those that did not (*P*=.008), whereas the opposite was true for health apps that reported including users during development (*P*=.02). No significant group differences were observed in user ratings. Finally, reported user input was associated with improved compliance with best practices across all examined areas: clinical assurance (eg, meeting ESF evidence requirements), data privacy (eg, including a statement that confirms the app’s compliance with the GDPR), risk management (eg, clearly stating who the app should or should not be used by), and user experience and app functionality (eg, allowing users to manage notification settings).

The interpretation of these findings in light of the previous literature is discussed in the following sections.

### User Input Differs Across Countries, Condition Areas, and App Risks

The percentage of mobile health apps that reported user input differed across countries. Interestingly, the countries in which higher percentages of apps with reported user input were observed tended to be those with higher digital maturity levels in health care [23]. Digital maturity in this context describes the extent to which a country can derive value from technology in a health care setting and was evaluated across three main areas in the cited report [23]: (1) initiatives related to digital health, including policies and funding availability, as a foundation for the country’s digital health care transformation; (2) infrastructure, including electronic health records, data standards, and interoperability, as a basis for interconnected systems and high-quality data; and (3) implementation efforts, including for telehealth, artificial intelligence, and internet-based studies, as a measure of the country’s ability to make use of digitally collected data to improve population health management [23]. Notably, the country with the highest digital maturity score in health care across Europe, the Middle East, and Africa (Denmark [23]) also exhibited by far the highest percentage of reported user inclusion during development in our study. Similar relationships between high digital maturity and reported user input in our study were observed for England and Sweden and, to a lesser extent, France and Belgium [23]. Thus, it may be the case that a better digital health infrastructure and more established processes make it easier for app developers to involve users. For instance, countries with higher, compared with those with lower, digital maturity in health care may offer better access to funding or know-how to support user involvement activities or may allow developers to invest more resources into those activities because of more streamlined and, thus, less resource-intensive processes in other areas such as for establishing interoperability. If this is the case, user involvement activities may become more widespread in other countries as their digital maturity in health care improves.

The percentage of mobile health apps that reported seeking user input during development or testing also differed across condition areas, with the highest percentages of user input being observed in the areas of oncology, diabetes, and cardiology. This finding is in line with 2 recent reviews, which reported that most papers on patient and public involvement in health care innovation were published in the fields of oncology and diabetes (as well as mental health [11,12]). Notably, the condition areas in which the highest percentage of user inclusion was observed in this study were those with the largest number of mobile health apps on the market (apart from mental health [24]). Thus, facing high levels of competition may encourage developers to involve users in the development process. In addition, the uneven distribution of funding across condition areas may also play a role as the aforementioned clinical indications were among the top 5 most funded digital health areas in 2022 [25], which may increase both the number of apps on the market and the availability of resources for user involvement activities in those areas. This highlights the need for digital health funding in other condition areas, including to cover costs of user involvement activities as a crucial part of the app development and testing process.

The third dimension across which the percentage of mobile health apps with reported user input differed was the NICE ESF tier. Specifically, there was a tendency of an increasing ESF tier being associated with a greater percentage of apps that reported seeking user input during development or testing. This pattern suggests that developers are aware that user inclusion is especially important for health apps with high risk. Furthermore, the results may indicate that developers require more user input to understand how best to communicate with users and what their needs are for apps that provide tailored advice on diagnosis and treatment (tier 3) than for simpler health apps (tiers 1 and 2). Moreover, developers of riskier health apps may be more likely to seek funding from organizations such as the National Institute for Health and Care Research, which includes cocreation of the app with patients as a core requirement. However, it should be noted that, even in ESF tier 3, fewer than half (105/263, 39.9%) of the apps included users during development, which underscores the need for further education and support of developers regarding the importance and execution of user involvement during the early stages of the life cycle.

### User Input During Testing Is Associated With More Downloads but Not With Higher User Ratings

When examining user ratings for mobile health apps that did and did not report seeking user input during development or testing, no significant differences were observed. This is somewhat surprising, especially in light of a recent study reporting that user experience was the most important determinant of positive health app user reviews [26]. However, the same study also observed that aspects such as payment problems and bugs or stability issues after updates were major factors that resulted in negative user reviews. Such issues are unlikely to arise during early user involvement (potentially working with free and low-fidelity app test versions), which may explain why no relationship between user ratings and user input during development was observed in this study. Moreover, the lack of relationship between user ratings and user input during testing could be partially due to user views only being taken into account during early testing and not at later stages as updates to the app are made [26]. Therefore, there may be a stronger relationship between user input and positive user ratings for earlier versions of the app, which may become weaker as updates are made without user contributions. We were unable to assess this suggestion using the available data, but if this was found to be the case, it would underline the importance of continued user involvement and consideration of user feedback (eg, from user reviews) at later life cycle stages.

Another surprising finding was that mobile health apps that did not report including users during development were downloaded more frequently than those that did. A potential explanation for this observation may be that there is a trade-off between resource investment for user involvement and marketing, with the latter being a stronger determinant of the number of downloads. In line with this suggestion, a recent review cited financial constraints as a barrier to patient and public involvement in digital health innovation [11]. In this context, it should also be noted that, although the number of initial downloads may not necessarily be related to user input, the *sustained* use, which ultimately determines health outcomes and the long-term success of the app, is likely to be linked more closely to user inclusion. Specifically, it has been argued that a lack of user involvement can give rise to barriers to sustained health app use, such as poor usability, failure to meet real-life user needs, and addition rather than elimination of effort in the health management process [27]. Moreover, it is worth noting that the hypothesized relationship between downloads and user input during *testing* was observed, with mobile health apps that did include users during testing demonstrating more downloads than those that did not. Notably, this study determined user input during testing based on publicly available information shown on app websites. Such information may be compelling to potential users, inducing them to download the app, which may explain the observed findings. This demonstrates the value to developers of conducting and showcasing user inclusion activities.

### User Input Is Associated With Improved Compliance With Best Practices Regarding Clinical Assurance, Data Privacy, Risk Management, and User Experience

Examining the relationship between quality characteristics and user inclusion revealed a significant positive association between reported user input and alignment with ESF tier clinical evidence requirements. At first sight, it may be expected that this effect is due to user input resulting in more engaging apps, which, in turn, may lead to better adherence and, thus, higher clinical efficacy and effectiveness of the health apps. However, this hypothesis was not confirmed in this study as no significant association was observed between user input and reported app effectiveness or efficacy. Alternatively, the observed relationship may be due to a third-variable effect, with developers who are more aware of best practices for app development being more likely to include users *and* to conduct appropriate, high-quality research studies that meet ESF tier requirements. This could be a valuable area for future research.

Another quality indicator that was found to be associated with reported user input was data privacy best practice, as indicated by explicit compliance with the GDPR and transparency about data processing. Again, this may be a third-variable effect because of an association between awareness of best practices for both data privacy and user involvement. Alternatively, the observed association may be due to users stressing the importance of data privacy during user involvement activities. Consistent with this suggestion, a recent review of attitudes toward the use of health data found that, although public and patient participants generally support data sharing for research purposes, many raised concerns about confidentiality breaches and potential abuses of the data [28]. Along similar lines, a number of reviews and frameworks for user involvement in research emphasize the importance of transparency regarding data handling [29-31]. Therefore, it is possible that developers with more transparent data-handling practices find it easier to recruit users for involvement activities. This suggestion is in line with reports that concerns about data privacy are a barrier to patient and public involvement in digital health innovation [11].

Significant positive associations were also observed between reported user input and the presence of risk management practices, such as clearly indicating who the app should not be used by and what risks are associated with using the app. It is likely that the need to clearly inform users of potential risks may be highlighted during user involvement activities, which may provide insights into risks beyond the developers’ initial expectations. This is likely the case as perceptions of risks and risk-benefit trade-offs can differ between developers and patients, who have firsthand experience of the condition being targeted by the app (which is why patient involvement in risk assessments is increasingly being considered an important factor for regulatory decisions regarding new health technologies [32-34]).

With regard to user experience, it was observed that reported user input was associated with a higher likelihood of the app allowing users to manage notifications, clinical terms being clearly explained, and the developer committing to addressing reported issues. Moreover, mobile health apps that involved users were also likely to include goal setting, gamification, or user connection (eg, forum) features. This finding is in line with previous research showing that aspects such as notification management and goal setting are important to users when they review health apps and decide whether to continue using them [26,35]. The observed association between the presence of these features and user input suggests that the decision to include these features may have arisen from user involvement activities, suggesting that user inclusion leads to improved adaption of features to user expectations.

### Related Work

There are numerous previous studies that have evaluated health apps according to different quality dimensions. These reviews have revealed that many health apps do not follow best practices in areas such as data privacy and security [36-39], clinical safety [40], or efficacy and effectiveness evidence [37,38,41].

In addition, studies more specifically focused on user engagement have shown that aspects such as visual design and engaging presentation of app content are correlated with key mobile health app metrics, namely, use time and 30-day retention rates [42]. As design- and engagement-related aspects can likely be improved by seeking user input during development and testing, this finding underscores the importance of user involvement, which is in line with this study.

Relatedly, 2 recent papers have highlighted the usefulness of considering (automatically analyzed) app store user reviews in identifying and addressing the challenges that users experience with health apps. Key issues reported by users included compatibility and log-in difficulties, stability and accessibility problems, and privacy-related concerns, which contributed to low star ratings on the app store [26,43]. In line with these observations, a pilot study examining human-centric issues with health apps across different countries found that accessibility, usability, and data privacy issues were regarded as essential points to consider by different stakeholders [44]. These findings emphasize the importance of considering user views both during initial development and after app updates to ensure a positive user experience.

### Summary of Implications for Practice and Recommendations for Future Work

Our research found that, even for high-risk apps, fewer than half of developers reported involving users during development. As mentioned previously, this highlights the need for further education and support for developers regarding the importance and implementation of user involvement during the early stages of the product life cycle. Building on existing work that revealed barriers to meaningful user involvement [11], a support program for developers could be devised. The effectiveness of this program could be evaluated through future research comparing user involvement practices between health app development companies (matched on various characteristics) that did and did not take part in the program.

Moreover, as discussed previously, there may be a relationship between funding availability and the willingness or ability of developers to involve users during development and testing. Future research (in collaboration with app developers) could examine this relationship more directly by assessing whether there is a significant association between investment in individual health apps and whether they seek user input or by determining what percentage of developers start involving users at which funding stage. If this research indicates that many developers are only willing to budget for user involvement activities once they have received a large amount of funding, this would suggest that it may be useful for investors to earmark some (earlier) funding for user involvement given the importance of considering user views to ensure the success of an app [26].

Furthermore, our findings indicate that seeking user input is associated with improved compliance with best practices across various domains. Therefore, *where no other guidance is available*, stakeholders such as patients and health care professionals looking for health apps may benefit from prioritizing apps that seek user input during development and testing as this may be a proxy for broader app quality.

### Strengths and Limitations

The limitations of this study should be noted. First, only countries and condition areas with >10 mobile health apps were included in the relevant data summaries as samples of <10 mobile health apps are likely not representative of the larger app “population” in a given country or condition area. Therefore, our findings may not be generalizable to excluded countries and conditions with not enough observations. Relatedly, results from countries and condition areas for which a relatively small number of apps were included in our sample should be interpreted with caution.

Secondly, user involvement and input was defined somewhat more broadly in this study than is common in the previous literature (see the *Determination of User Input* section). This was because the study relied on a data set that had been collected for practical assessment purposes rather than specifically for research. Although this limitation should be kept in mind when interpreting the study findings, it is encouraging to see that there is convergence between the observations of this study and those of previous research (as described in the previous sections) that used a stricter definition of user or patient and public involvement.

Third, the determination of the presence of user input in this study relied on information from within the app and associated websites. It is possible that, in some cases, user involvement did take place without this being publicly indicated and, thus, without it being taken into account in this study. However, given that user involvement is generally regarded as a strength, it seems unlikely that (many) developers would forego the opportunity to publicly state that they took users’ input into account.

Finally, it should be noted that no information was available regarding the quality and meaningfulness of the user involvement activities or who the involved users were (eg, whether they were representative of the actual users and whether they included minority groups or individuals from different socioeconomic backgrounds and with different levels of digital literacy—this will be examined in future versions of the ORCHA assessment). It was also not recorded what user involvement methods or levels of involvement were applied (eg, whether co-design or consultation was used). It is possible that findings may differ depending on the “depths” and quality of user involvement, which would be an interesting area of examination for future research.

Apart from these limitations, a major strength of this study is the use of a unique data set with detailed assessment information for >1500 mobile health apps across different countries and condition areas. This allowed the study to generate findings beyond those that could be gathered from the peer-reviewed literature, which is important given that likely not all user involvement activities are published in academic articles. Moreover, to our knowledge, this is the first study to examine the relationship between user input and compliance with best practices across the domains of clinical assurance, data privacy, risk management, and user experience.

### Conclusions

In summary, this study found that the prevalence of user input during mobile health app development or testing differed across countries, condition areas, and ESF tiers. The countries and condition areas in which the highest percentage of health apps with user input were observed tended to be those with higher digital maturity in health care and more funding availability, respectively. This suggests that there may be a trade-off between developers’ willingness or ability to involve users during development or testing and the need to meet challenges arising from infrastructure limitations and financial constraints. Moreover, the finding of a positive association between user input and compliance with best practices indicates that, *where no other guidance is available*, users may benefit from prioritizing mobile health apps that involved users during development and testing as this may be a proxy for broader app quality.

## Appendix

Multimedia Appendix 1 Conversion of download number ranges to download levels.

## Abbreviations

ESF: Evidence Standards Framework
GDPR: General Data Protection Regulation
NICE: National Institute for Health and Care Excellence
OBR: Organisation for the Review of Care and Health Apps Baseline Review
ORCHA: Organisation for the Review of Care and Health Apps
