# Supplementary material for: Association Between the Characteristics of mHealth Apps and User Input During Development and Testing: Secondary Analysis of App Assessment Data
Source: JMIR Mhealth Uhealth. 2023 Nov 22;11:e46937. doi: 10.2196/46937 (PMC10701645; doi:10.2196/46937)
Supplement: Multimedia Appendix 1 [file mhealth_v11i1e46937_app1.docx]

Multimedia Appendix 1

Table S1: Indication of which download level was ascribed to which download number range obtained from the app store, and the number of apps falling into each range and level. Note that download numbers were only available from the Google Play Store (for Android apps; n = 777) and not from the iOS App Store.

| **Download number range** | **Download level** | **Number of apps falling into the range/level** |
| --- | --- | --- |
| 100,000,000 and above | 17 | 3 |
| 50,000,000 - 99,999,999 | 16 | 6 |
| 10,000,000 - 49,999,999 | 15 | 47 |
| 5,000,000 - 9,999,999 | 14 | 29 |
| 1,000,000 - 4,999,999 | 13 | 59 |
| 500,000 - 999,999 | 12 | 51 |
| 100,000 - 499,999 | 11 | 106 |
| 50,000 - 99,999 | 10 | 56 |
| 10,000 - 49,999 | 9 | 129 |
| 5,000 - 9,999 | 8 | 56 |
| 1,000 - 4,999 | 7 | 107 |
| 500 - 999 | 6 | 31 |
| 100 - 499 | 5 | 53 |
| 50 - 99 | 4 | 17 |
| 10 - 49 | 3 | 18 |
| 5 - 9 | 2 | 3 |
| 1 - 4 | 1 | 4 |
| 0 | 0 | 2 |
